# Functional Study of PTSMAD4 in the Spermatogenesis of the Swimming Crab *Portunus trituberculatus*

**DOI:** 10.3390/ijms252313126

**Published:** 2024-12-06

**Authors:** Yu Zhang, Qiu-Meng Xiang, Chang-Kao Mu, Chun-Lin Wang, Cong-Cong Hou

**Affiliations:** Key Laboratory of Aquacultural Biotechnology, Key Laboratory of Marine Biotechnology of Zhejiang Province, College of Marine Sciences, Ningbo University, Ningbo 315211, China; 2211130126@nbu.edu.cn (Y.Z.); 1911091064@nbu.edu.cn (Q.-M.X.); muchangkao@nbu.edu.cn (C.-K.M.); wangchunlin@nbu.edu.cn (C.-L.W.)

**Keywords:** crustacean, TGF-β, PTSMAD4, spermatogenesis, *Portunus trituberculatus*

## Abstract

*Portunus trituberculatus* holds significant economic value. The spermatogenesis is regulated by numerous signaling pathways. Among them, the TGF-β signaling pathway plays an important role in the development of testes and spermatogenesis. Smad4 is a Co-Smad protein that forms a complex with R-Smad to regulate the expression of target genes. The sperm structure in crustaceans differs greatly from that in mammals, with mature sperm lacking tails. Our previous studies have reported the function of R-Smad in the spermatogenesis of *P. trituberculatus*. In this study, we cloned the full-length cDNA sequence of *PTSMAD4*; immunofluorescence technology revealed that PTSMAD4 is expressed throughout all stages of spermatogenesis. We knocked down the expression of PTSMAD4 in *P. trituberculatus* using RNAi technology, and the immunofluorescence results show abnormal co-localization and weakened signals of PTSMAD4 and PTSMAD2. Additionally, transcriptome sequencing results enriched functional genes and pathways related to spermatogenesis. This study indicates that PTSMAD4 may participate in the spermatogenesis process through its involvement in signal transduction. This research not only lays the foundation for further study of the function of the TGF-β signaling pathway in spermatogenesis but also provides a theoretical basis for further investigation of the spermatogenesis mechanism in crustaceans.

## 1. Introduction

Spermatogenesis is a highly complex and orderly physiological process that is divided into three main stages: first, spermatogonial cells differentiate into spermatocytes through mitosis; next, spermatocytes undergo meiosis to form spermatids; and finally, spermatids further differentiate during spermiogenesis to ultimately become mature sperm [1]. In mammals, spermatogenesis involves the condensation and morphological changes of spermatocytes, including the discarding of the cytoplasm, the formation of the acrosome and tail, and eventually the development into mature sperm with tails [2,3]. In contrast, crustaceans develop structurally unique tailless sperm during spermatogenesis [4]. The swimming crab (*Portunus trituberculatus*), as a species of significant economic value among crustaceans, has received considerable attention for its reproductive biology. Therefore, studying the molecular mechanisms of its spermatogenesis process is of great importance for providing a theoretical basis for the selection and breeding of high-quality larvae [5].

Spermatogenesis is a physiological process delicately regulated by multiple signaling pathways [6], including the PI3K/AKT signaling pathway [7], Wnt/β-catenin signaling pathway [8], and transforming growth factor-β (TGF-β) signaling pathway [9,10]. The TGF-β signaling pathway is a crucial intracellular signal transduction mechanism that plays an essential role in cell proliferation, differentiation, gonadal development, and spermatogenesis [11,12,13,14,15,16]. In epithelial cells, TGF-β can induce epithelial–mesenchymal transition (EMT), endowing epithelial cells with the characteristics of mesenchymal cells [17,18]. In rat granulosa cells, TGF-β acts as a proliferation regulator, mediating estrogen-induced granulosa cell growth [19]. In vertebrates, the loss of genes related to the TGF-β signaling pathway can lead to impaired spermatocyte development and disrupted spermatogenesis [20], thereby affecting sperm quality [21]. For instance, during the gonadal differentiation process in medaka fish, the activation of the *GSDF* gene in the TGF-β signaling pathway can initiate testicular differentiation [22]. In the Chinese mitten crab (*Eriocheir sinensis*), the inhibition of es-bmp2 during spermatogenesis results in abnormal sperm nuclear morphology [23].

The TGF-β signaling pathway involves various ligands, receptors, and Smad proteins. TGF-β ligands bind to TGF-β receptors on the cell membrane, activate the receptors, and transmit signals to R-Smad proteins, which ultimately form a complex with Co-Smad and enter the nucleus to regulate gene expression [24,25]. The Smad protein is a key protein in the TGF-β signaling pathway. The Smad protein involved in the TGF-β signaling pathway was found in Drosophila melanogaster [16], and then its homologous protein Sma was found in nematode [14], and then related proteins were also found in humans and other animals. So, involved in TGF-β, these proteins for signal transduction are named Smad proteins. The Smad protein family, crucial in the TGF-β signaling pathway, is divided into three types based on structure and function: Co-Smad, I-Smad, and R-Smad [22]. I-Smad exerts a negative regulatory effect on the pathway, while R-Smad, once activated by the receptor, combines with Co-Smad and enters the nucleus to regulate gene expression [13]. R-Smad includes Smad1, Smad2, Smad3, Smad5, and Smad8, with the Smad1/5/8 pathway typically activated by BMP/GDF ligands and the Smad2/3 pathway activated by TGF-β/activin/inhibin ligands [15]. In rat testes, Smad2 and Smad3 proteins are primarily located in the cytoplasm of germ cells, Sertoli cells, and testicular interstitial cells, assisting in the function of TGF-β signaling in spermatogenesis [26]. Smad4, as the sole Co-Smad, must form a complex with R-Smad to enter the nucleus and regulate gene transcription [27,28]. Smad4 is a gene that negatively regulates gene transcription and helps maintain vascular homeostasis and bone homeostasis [29,30,31]. In mouse germ cells, the knockout of Smad4 results in impaired testis development and reduced sperm production [32]. In pigs, Smad4 and Smad2 are expressed throughout the stages of follicular development, serving as key regulatory factors for ovarian development and oocyte growth [33]. In avian testes, Smad4 is widely expressed and participates in TGF-β signal transduction during spermatogenesis [34]. The above studies indicate that the TGF signaling pathway is involved in spermatogenesis in vertebrates and Smad4 has the function of regulating gene transcription; its function in crustaceans remains unclear.

Our team has demonstrated SMAD2 in R-Smad in previous studies; it may be involved in regulating the expression of genes related to spermatogenesis in swimming crab and may be involved in the formation of acrosome [35]. However, it is not yet clear whether Smad4 is involved in spermatogenesis and or participates in TGF-β signal transduction by combining with SMAD2. Therefore, this study aims to explore the function of Smad4 during spermatogenesis in the swimming crab. First, we cloned the full-length cDNA sequence of PTSMAD4 from the testis and studied its expression distribution in various tissues and germ cells, as well as the abundance distribution of PTSMAD4 and PTSMAD2 during the stages of spermatogenesis. By using RNAi technology to knock down the expression of PTSMAD4, we further clarified the involvement of PTSMAD4 in combination with PTSMAD2 in the spermatogenesis process. In addition, by employing transcriptome sequencing, we screened for genes and signaling pathways related to spermatogenesis, regulated by the TGF-β signaling pathway.

## 2. Results

### 2.1. Full-Length cDNA Sequence and Protein Structure Analysis of PTSMAD4

**Figure 1:**
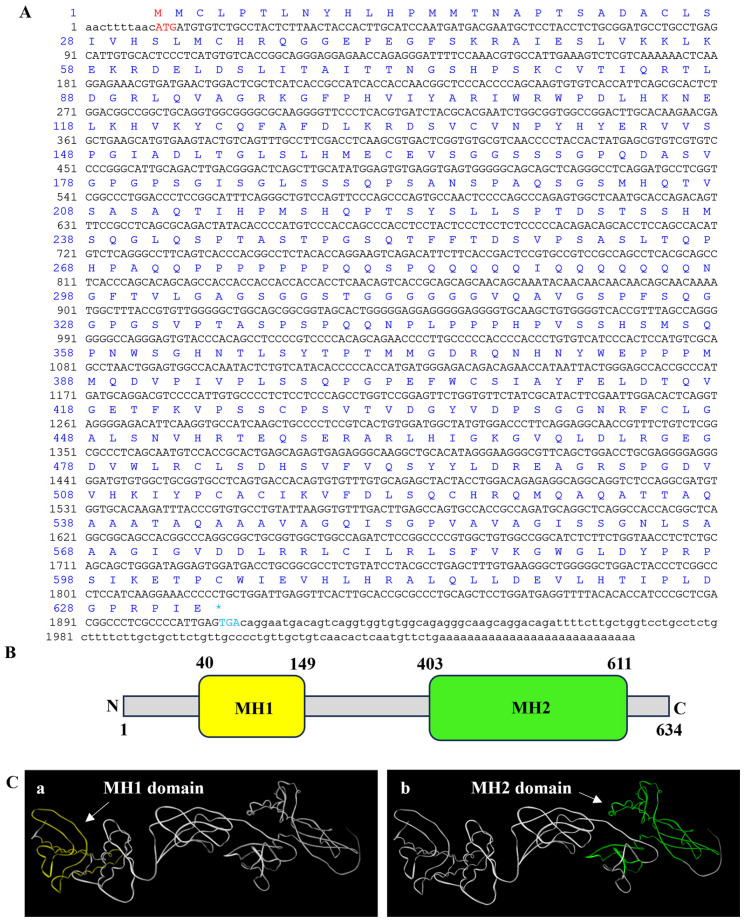
Full-length cDNA sequence and structural prediction of PTSMAD4. (**A**) The full-length cDNA sequence of PTSMAD4 and its corresponding amino acids, with the start codon ATG marked in red and the stop codon TGA in blue. “*” indicates the stop codon. (**B**) The secondary structure of the PTSMAD4 protein, with the MH1 domain (40–149 aa) in yellow and the MH2 domain (403–611 aa) in green. (**C**) The tertiary structure of the PTSMAD4 protein, with (**a**) indicating the MH1 domain and (**b**) indicating the MH2 domain.

The PTSMAD4 sequence was uploaded to NCBI (GenBank: PQ303701). The full-length cDNA sequence of *PTSMAD4* is 2059 bp, including a coding region of 1806 bp (encoding 601 amino acids), a 5′ untranslated region of 10 bp, and a 3′ untranslated region of 147 bp (Figure 1A). The protein has a molecular weight of 67.86 kDa and an isoelectric point of 7.13. The structural domains of PTSMAD4 were predicted using online tools SMART and CD-Search, revealing that PTSMAD4 consists of an N-terminal MH1 domain (40–149 aa) and a C-terminal MH2 domain (403–611 aa) (Figure 1B,C).

### 2.2. Multiple-Sequence Alignment and Phylogenetic Tree Analysis of PTSMAD4

**Figure 2:**
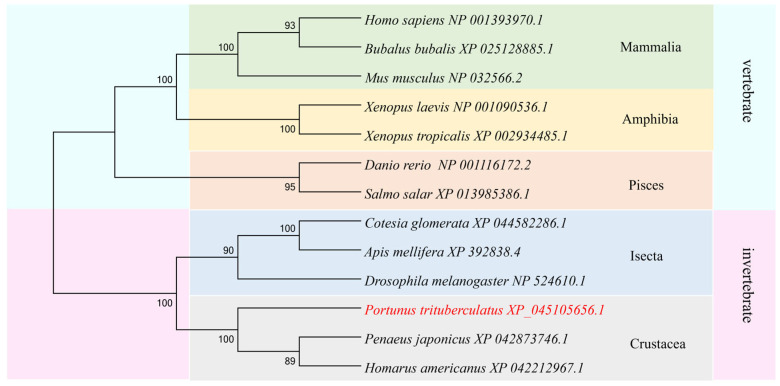
Phylogenetic tree of PTSMAD4. Red marks the swimming crab *P. trituberculatus*. PTSMAD4 is closely related to the crustacean Homarus americanus and Penaeus japonicus but is distantly related to vertebrates.

Multiple-sequence alignment was conducted on the amino acid sequences of the PTSMAD4 protein and its homologs from other species. The results show that the similarities of PTSMAD4 to the homologous proteins in humans (*Homo sapiens*), mice (*Mus musculus*), water buffalo (*Bubalus bubalis*), zebrafish (*Danio rerio*), African clawed frogs (*Xenopus laevis*), tropical clawed frogs (*Xenopus tropicalis*), American lobsters (*Homarus americanus*), and Japanese shrimp (*Penaeus japonicus*) were 63.7%, 63.0%, 63.5%, 62.9%, 64.1%, 62.2%, 62.4%, 78.5%, and 76.3%. The MH1 and MH2 domains of PTSMAD4 are highly conserved evolutionarily (Supplementary Figure S1). Phylogenetic tree analysis indicated that PTSMAD4 forms a clade with the American lobster (*Homarus americanus*) and Japanese shrimp (*Penaeus japonicus*) from the crustacean group, indicating a closer phylogenetic relationship, while it is more distantly related to vertebrates (Figure 2).

### 2.3. Expression Characteristics of PTSMAD4 mRNA in Various Tissues

**Figure 3:**
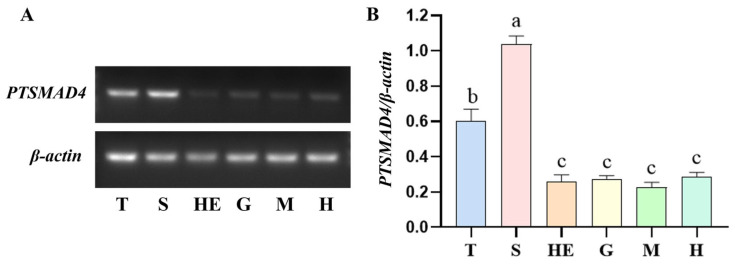
Expression of *PTSMAD4* mRNA in different tissues of the swimming crab *P. trituberculatus*. (**A**) Semi-quantitative PCR results of *PTSMAD4* mRNA in different tissues of the swimming crab. (**B**) Grayscale values were extracted using Image J V1.8.0.112 software, and the expression of *PTSMAD4* mRNA in each tissue was analyzed with β-actin as the internal reference gene. T: testis; S: vas deferens; HE: hepatopancreas; G: gills; M: muscle; H: heart. Different lowercase letters indicate significant differences, *p* < 0.001.

To investigate the expression of *PTSMAD4* mRNA in various tissues of the swimming crab *P. trituberculatus*, we examined the expression levels of *PTSMAD4* mRNA in the testes, vas deferens, heart, muscle, gills, and hepatopancreas of the crab (Figure 3). The results show that *PTSMAD4* mRNA is widely expressed across all tissues, with higher (*p* = 0.00075) expression in the vas deferens than testes, and both than the expression in muscle (*p* = 0.00093 in testis vs. muscle, *p* = 0.000012 in vas deferens vs. muscle).

### 2.4. Preparation and Specificity Testing of PTSMAD4 Antibody

To investigate the function of the PTSMAD4 protein in the spermatogenesis process of the swimming crab *P. trituberculatus*, we obtained the recombinant PTSMAD4 protein through prokaryotic expression. The Coomassie Brilliant Blue staining results show that the protein size was consistent with the predicted size of 29.4 kDa (Supplementary Figure S2A). New Zealand rabbits were immunized periodically, and polyclonal rabbit antibodies were obtained. Western blot testing was used to assess the specificity of the antibodies, and the results indicate that the anti-PTSMAD4 rabbit antibody had good specificity, with a single band matching the predicted molecular weight of the PTSMAD4 protein, 67.86 kDa (Supplementary Figure S2B).

### 2.5. Abundance and Distribution of PTSMAD4 in the Spermatogenesis of the Swimming Crab P. trituberculatus

**Figure 4:**
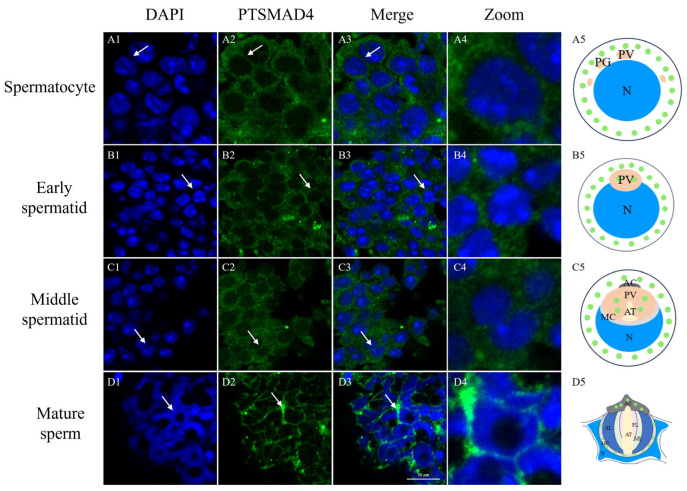
Abundance and distribution of the PTSMAD4 protein during spermatogenesis in *P. trituberculatus*. Schematic diagrams of the PTSMAD4 protein (**A5**,**B5**,**C5**,**D5**). The DAPI signal represents the cell nucleus and represents sperm cells at different stages. PTSMAD4 signals represent the abundance distribution of PTSMAD4 at different stages of spermatogenesis in the swimming crab. Panels (**A1**–**A4**) represent spermatocytes; (**B1**–**B4**) represent early spermatids; (**C1**–**C4**) represent mid-stage spermatids; (**D1**–**D4**) represent mature sperm. The arrow points to the cells at each stage. The zoom represents the enlarged image of a sperm cell, pointed out by a white arrow. N: nucleus; PV: pro-acrosome vesicle; AT: acrosome tube; AC: acrosome cap; MC: membrane complex; FL: fibrous layer; ML: middle layer; SL: stratified layer. Scale bar = 10 μm.

Immunofluorescence was used to detect the abundance and distribution of the PTSMAD4 protein during spermatogenesis in the swimming crab *P. trituberculatus*. As shown in Figure 4, the PTSMAD4 protein can be observed throughout the spermatogenesis process. In spermatocytes and early spermatids, the PTSMAD4 signal is uniformly distributed in the cytoplasm of early spermatids (Figure 4(A1–B3)). In mid-stage spermatids, it accumulates on the inner side of the gradually forming cup-shaped cell nucleus (Figure 4(C1–C3)). In mature sperm, it is mainly distributed around the cell nucleus (Figure 4(D1–D3)). The results showed that the deformation of the PTSMAD4 around the nucleus during spermatogenesis suggests that PTSMAD4 may be involved in the nuclear deformation process in spermatogenesis of the swimming crab *P. trituberculatus*.

### 2.6. Knockdown of PTSMAD4 In Vivo

#### 2.6.1. Detection of Knockdown Efficiency

**Figure 5:**
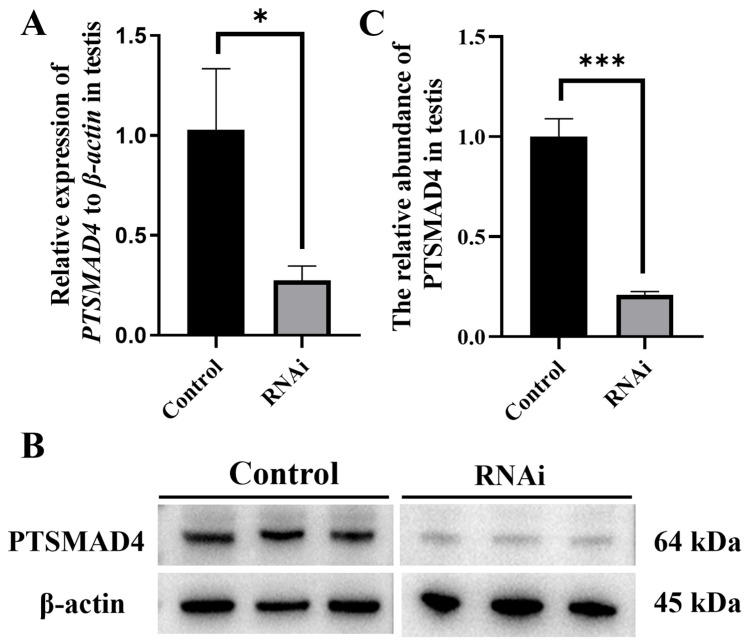
Detection of interference efficiency after dsRNA interference. (**A**) qPCR detection of *PTSMAD4* mRNA expression in the testes after dsRNA interference. (**B**) Western blot (WB) detection of PTSMAD4 abundance in the testes before and after interference. (**C**) Extraction of protein band grayscale values using Image J V1.8.0.112 software. “*”, *p <* 0.05, *p* = 0.0144, “***”, *p* < 0.001, *p* = 0.0001.

To further investigate the function of PTSMAD4 in the spermatogenesis of the swimming crab *P. trituberculatus*, we synthesized the dsRNA of PTSMAD4 to knock down its expression. The knockdown efficiency was detected using qPCR, and the results show an efficiency of 73.3% (Figure 5A). Total proteins were extracted from the testes of the swimming crab, and the protein abundance after knockdown was detected using a Western blot (WB) (Figure 5B). The protein band after knockdown was significantly weaker compared to the control group (*p* = 0.0001). The grayscale values were extracted using Image J V1.8.0.112, and the results show that the level of PTSMAD4 protein was also significantly reduced, with a knockdown efficiency of 79% (Figure 5C), indicating that the knockdown experiment was successful.

#### 2.6.2. The Abundance and Distribution of PTSMAD4 in the Spermatogenesis of the Swimming Crab *P. trituberculatus*

**Figure 6:**
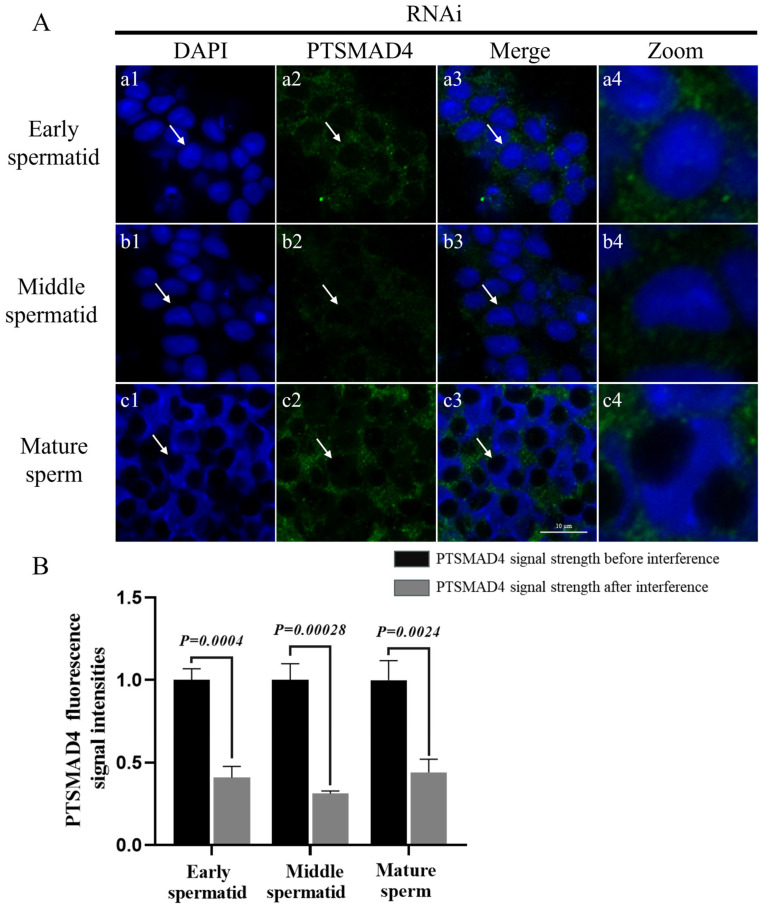
(**A**) Abundance and distribution of PTSMAD4 in the spermatogenesis of the swimming crab *P. trituberculatus* after interference. The DAPI signal represents the cell nucleus and represents sperm cells at different stages. PTSMAD4 signals represent the abundance distribution of PTSMAD4 at different stages of spermatogenesis in the swimming crab. (**a1**–**a4)** are early spermatids; (**b1**–**b4**) are mid-stage spermatids; (**c1**–**c4**) are mature sperm. The arrow points to the cells at each stage. The zoom represents the enlarged image of a sperm cell, pointed out by a white arrow. Scale bar = 10 μm. (**B**) PTSMAD4 fluorescence signal intensities.

Immunofluorescence detected the abundance and distribution of PTSMAD4 at various stages after interference, showing a significant reduction in PTSMAD4 signaling across all stages of spermatogenesis (Figure 6).

### 2.7. The Abundance and Distribution of PTSMAD4 and PTSMAD2 in the Spermatogenesis of the Swimming Crab P. trituberculatus

**Figure 7:**
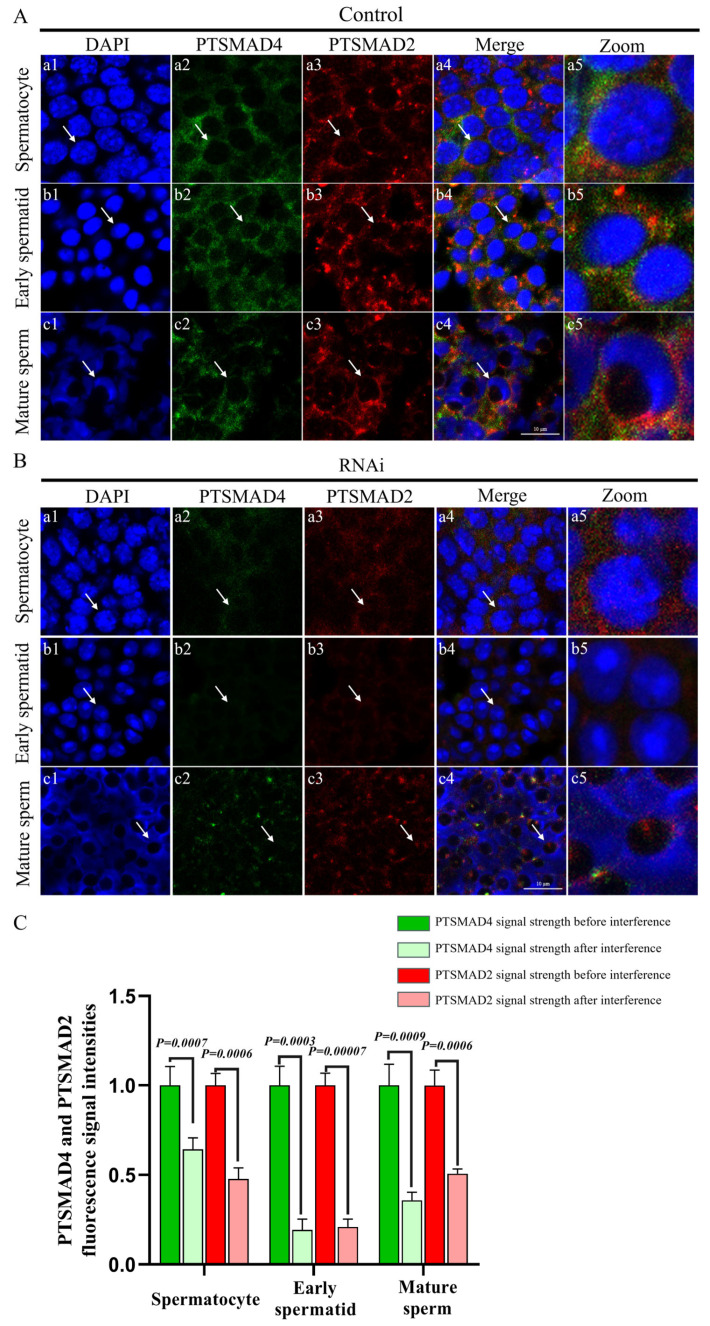
Abundance and distribution of PTSMAD4 and PTSMAD2. (**A**) Changes in the abundance and distribution of PTSMAD4 and PTSMAD2 before interference. (**B**) Changes in the abundance and distribution of PTSMAD4 and PTSMAD2 after interference. The DAPI signal represents the cell nucleus, and the green fluorescence signal is for PTSMAD4; the red signal is for PTSMAD2. Panels (**a1**–**a5**) represent spermatocytes; (**b1**–**b5**) represent early spermatids; (**c1**–**c5**) represent mature sperm. The arrow points to the cells at each stage. The zoom represents the enlarged image of a sperm cell, pointed out by a white arrow. Scale bar = 10 μm. (**C**) PTSMAD4 and PTSMAD2 fluorescence signal intensities.

**Figure 8:**
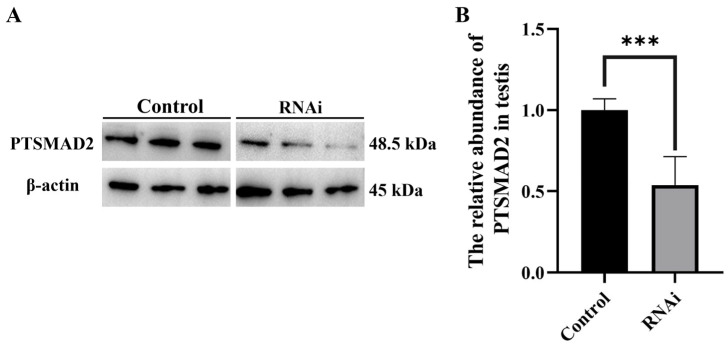
Protein abundance PTSMAD2 after knockdown PTSMAD4. (**A**) Western blot (WB) detection of PTSMAD2 abundance in the testes before and after interference. (**B**) Extraction of protein band grayscale values using Image J V1.8.0.112 software., “***” *p* < 0.001, *p =* 0.0006.

Immunofluorescence was used to detect the abundance and distribution of PTSMAD4 and PTSMAD2. In spermatocytes and early spermatids, the signals of PTSMAD4 and PTSMAD2 were uniformly distributed in the cytoplasm, with co-localization observed. However, in mature sperm, PTSMAD4 was localized to the perinuclear cytoplasm, while the PTSMAD2 protein was found in the acrosome tube and acrosome cap, showing no co-localization. After knocking down PTSMAD4, compared to the control group (Figure 7A,C), the signals in spermatocytes and early spermatids distributed in the cytoplasm were weakened, and co-localization was abnormal. In mature sperm, PTSMAD4 was still localized to the perinuclear cytoplasm, but the PTSMAD2 protein aggregated in the acrosome tube and acrosome cap, with weakened signals, and the two did not co-localize (Figure 7B,C). We measured the abundance of PTSMAD2 proteins after knockout PTSMAD4, and the results showed that the abundance of PTSMAD2 proteins was significantly reduced (Figure 8), further demonstrating that PTSMAD4 interacts with PTSMAD2 and PTSMAD4. Synergistically, PTSMAD2 is involved in the spermatogenesis of the swimming crab *P. trituberculatus*.

### 2.8. Transcriptome Analysis

#### 2.8.1. Differential Gene Expression Analysis

**Figure 9:**
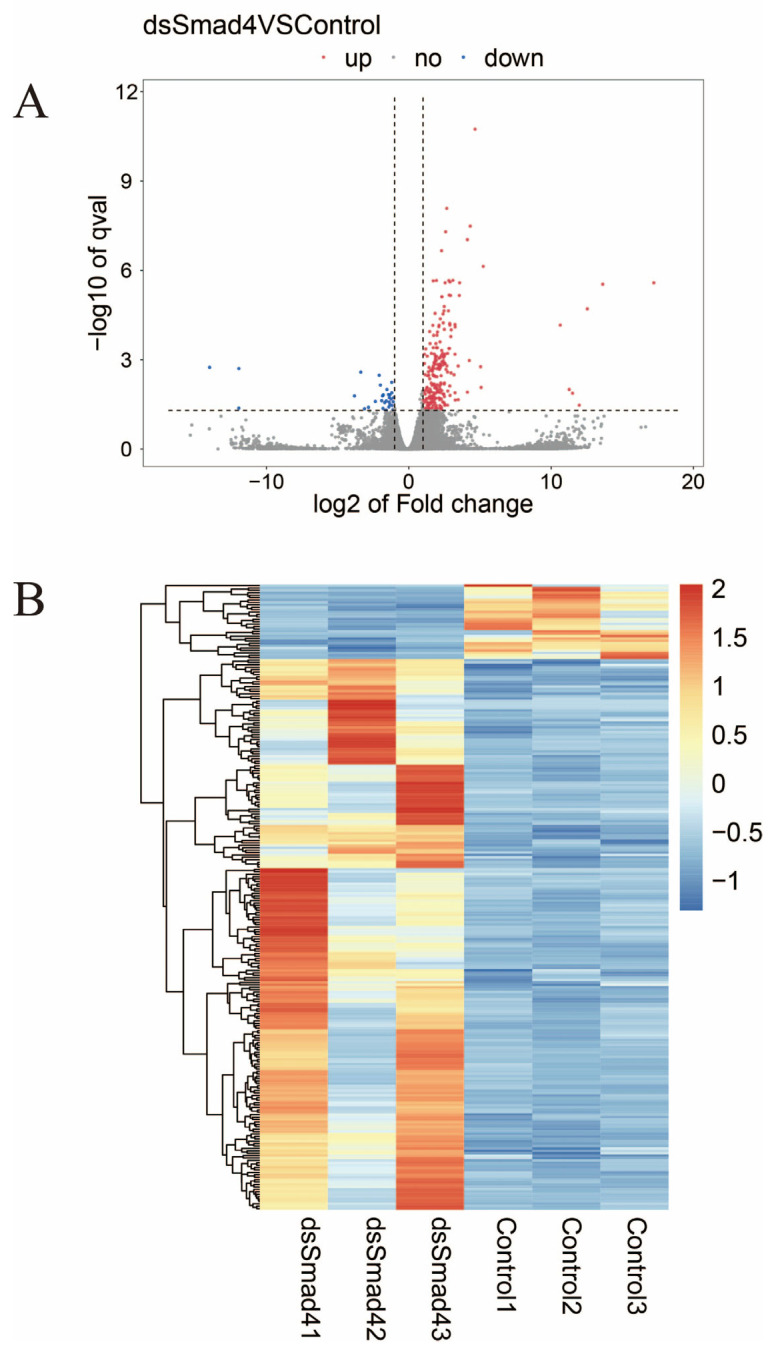
Differential gene expression analysis. A volcano plot was drawn for all genes in the differential expression analysis with log2(fold change) on the *x*-axis and −log10(*q*-value) on the *y*-axis (**A**). Red dots represent upregulated genes, blue dots represent downregulated genes, and gray dots represent genes with non-significant differences. To more intuitively display the expression patterns of the differentially expressed genes, a heatmap was created with samples on the *x*-axis and differentially expressed genes on the *y*-axis (**B**). In (**B**), each column represents a sample, each row represents a gene, and different colors indicate different levels of expression.

Differential expression analysis was performed on the transcriptome sequencing results to identify genes with altered expressions before and after the knockdown of PTSMAD4. The results indicate that after the knockdown of PTSMAD4, a total of 260 differentially expressed genes were identified in the testes of the swimming crab, of which 229 were significantly upregulated genes and 31 were significantly downregulated genes (Figure 9).

#### 2.8.2. GO Enrichment Analysis

**Figure 10:**
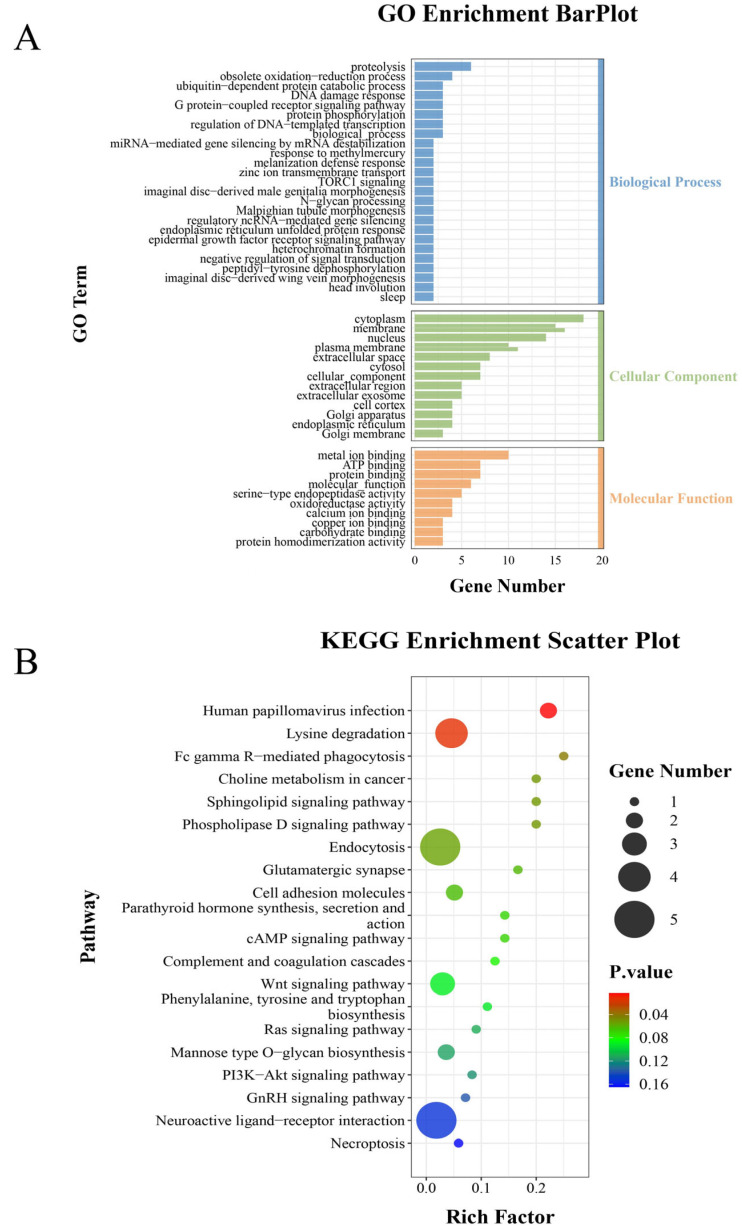
(**A**) The *x*-axis represents the number of differential genes enriched in GO terms, and the *y*-axis represents the GO terms. (**B**) The *x*-axis represents the proportion of differential genes in the pathway relative to the total number of genes in the pathway; the larger the value, the higher the enrichment degree; the *y*-axis is the KEGG signaling pathway; the size of the bubble indicates the number of genes, and the color of the bubble represents the significance of the enrichment analysis—the smaller the value, the more significant the enrichment.

To conduct an in-depth study of differentially expressed genes, GO enrichment analysis was performed on the differential genes between the PTSMAD4 interference group and the control group. Among the significantly enriched GO categories, the biological process (BP) includes proteolysis, protein phosphorylation, G protein-coupled receptor signaling pathway, etc. The cellular component (CC) includes cytoplasm, nucleus, Golgi apparatus, endoplasmic reticulum, etc.; the molecular function (MF) includes metal ion binding, ATP binding, protein binding, etc. (Figure 10A). Among these, proteolysis, Golgi apparatus, and protein phosphorylation are closely related to spermatogenesis.

#### 2.8.3. KEGG Enrichment Analysis

To further investigate the pathways involved in differentially expressed genes, a KEGG pathway enrichment analysis was conducted on the identified differentially expressed genes. Pathways closely related to spermatogenesis include lysine degradation, the cAMP signaling pathway, the Wnt signaling pathway, the phosphatidylinositol 3-kinase-protein kinase pathway (PI3K-Akt signaling pathway), the GnRH signaling pathway, etc. (Figure 10B).

#### 2.8.4. RT-qPCR Validation of RNA-Seq

**Figure 11:**
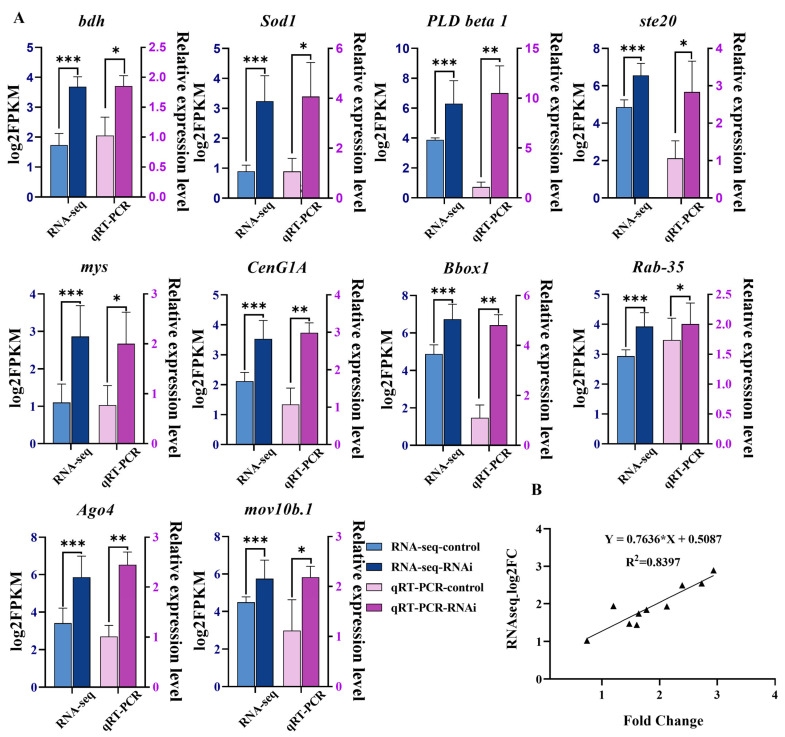
(**A**) The relative expression levels of 10 genes were analyzed by transcriptome sequencing and qPCR. (**B**) Correlation analysis between transcriptome data and qPCR validation. qPCR results were highly consistent with transcriptome results (R^2^ = 0.8397, n = 3). “*” *p* < 0.01; “**” *p* < 0.01; “***” *p* < 0.001.

We selected 10 differential genes for RT-qPCR, including *bdh*, *Sod1*, *PLD beta1*, *ste20*, *mys*, *CenG1A*, *Bbox1*, *Rab-35*, *Ago4*, and *mov10b.1*. The results show that the relative expression of genes using RT-qPCR and RNA-Seq was generally consistent (Figure 11A), and the Pearson correlation coefficient between the gene expression results of RT-qPCR and RNA-Seq was 0.8397 (Figure 11B), confirming the reliability of the Illumina sequencing.

## 3. Discussion

### 3.1. Structural Features and Expression Distribution of PTSMAD4

Smad4 is one of the core members of the TGF-β signaling pathway, which assists *Gallus domesticus* in transferring signals from the cell membrane to the nucleus and participates in the regulation of gene expression [34]. To explore the function of PTSMAD4 in the spermatogenesis of the swimming crab *P. trituberculatus*, we first cloned the full-length sequence of PTSMAD4 in the testis, and the results show that the PTSMAD4 protein has an MH1 domain and an MH2 domain. In human studies, the MH1 domain has been proven to specifically bind to DNA [36], while the MH2 domain is responsible for binding to receptors and participating in the transcription among Smad proteins [37,38]. Through multiple-sequence alignments, we found that the MH1 and MH2 domains of the PTSMAD4 protein are highly conserved compared to other species, so we speculate that PTSMAD4 also has the function of binding to specific DNA and receptors and participating in the transcription between Smad proteins. We detected the expression of *PTSMAD4* mRNA in different tissues and found that *PTSMAD4* mRNA was widely expressed in various tissues of the swimming crab, especially in the vas deferens and testes. We also studied the abundance and distribution of the PTSMAD4 protein during the stages of spermatogenesis and found that the PTSMAD4 protein is expressed in all stages and evenly distributed in the cytoplasm. In previous studies, Smad4 was expressed to varying degrees in the spermatogonia and spermatocytes of mice, which help in the transmission of signals in the TGF-β signaling pathway [39]. In dogs, Smad4 is widely expressed, mainly located in the interstitial cells and Sertoli cells of mature testes, and participates in the development of the testes and the spermatogenesis [40]. Based on our findings and the existing literature, we speculate that PTSMAD4 is involved in the transmission of signals in the TGF-β signaling pathway, as well as in the development of the testes and spermatogenesis in the swimming.

### 3.2. PTSMAD4 Collaborates with PTSMAD2 in Spermatogenesis

Smad4 and Smad2 are the main effector molecules of the TGF-β signaling pathway. Smad4 combines with the receptor-phosphorylated Smad2 to form a complex and enter the nucleus for transcriptional regulation, participating in the transcriptional regulation within the nucleus [37,41]. In the early embryonic development of the African clawed frog, Smad4 forms a complex with the induced phosphorylated Smad2 to participate in transcriptional activation and plays an important role in regulating TGF-β signal transduction [42]. In human cells, if the interaction between Smad4 and Smad2/3 is disrupted, TGF-β signal transduction will be inhibited, indicating that the interaction between Smad4 and Smad2/3 affects TGF-β signal transduction [43]. In previous studies, we explored the function of PTSMAD2 in spermatogenesis in the swimming crab [35], but it is not clear whether PTSMAD4 participates in spermatogenesis by combining with PTSMAD2. In this study, we detected the abundance and distribution of PTSMAD2 and PTSMAD4 proteins during spermatogenesis. The results show that both were collocated and evenly distributed in the cytoplasm of spermatocytes and early spermatids. However, PTSMAD4 in mature sperm was located in the cytoplasm around the nucleus and no longer was collocated with PTSMAD2, which remained in the cytoplasm. Therefore, we speculate that PTSMAD4 may assist PTSMAD2 in regulating gene expression during meiosis and the early stages of sperm formation, but PTSMAD4 may function independently in the later stages of sperm formation and in mature sperm. After knocking down PTSMAD4, the signals of PTSMAD4 and PTSMAD2 were weakened in all stages, the co-localization in spermatocytes and early spermatids was weakened, and the two were not co-localized in mature sperm. Therefore, we speculate that PTSMAD4 may cooperate with PTSMAD2 in regulating gene expression during meiosis and the early stages of sperm formation to participate in the process of TGF-β signal transduction, thereby participating in spermatogenesis.

### 3.3. PTSMAD4 Participates in Gene Regulation and Signal Transduction in Spermatogenesis

As an important regulatory factor of the TGF-β signaling pathway, Smad4 participates in signal transduction by mediating the transcriptional activation of target genes in mice and sheep [44,45,46,47]. To further explore the various regulatory mechanisms that PTSMAD4 may be involved in during spermatogenesis in the swimming crab, we conducted a comparative transcriptome analysis of the testis tissue of the swimming crab before and after knocking down PTSMAD4. Among the differentially expressed genes, we randomly selected 10 differential genes and verified the validity of the transcriptome data through qPCR.

In the GO category, we enriched biological processes such as protein hydrolysis and protein phosphorylation (biological process, BP). In recent years, in breast cancer cells, the TGF-β signaling pathway has been found to be involved in proteolysis, and the process of protein degradation is crucial in spermatogenesis [48,49]. In humans, sperm fertilization is related to the gradual phosphorylation of proteins [50]. We also enriched cellular components such as the Golgi apparatus and cytoplasm, as well as molecular functions such as metal ion binding and calcium ion binding (molecular function, MF). In mice, the Golgi apparatus is involved in the formation of the acrosome and is crucial for the transport process of spermatogenesis [51]. Calcium ion (Ca^2+^) channels play an important role in regulating human sperm vitality and are involved in various cellular signaling pathways [52]. These results suggest that PTSMAD4 may be involved in gene regulation and may participate in or regulate processes such as protein hydrolysis and protein phosphorylation, which are involved in material transport, acrosome formation, and the regulation of sperm vitality during spermatogenesis. In order to explore the signaling pathways associated with spermatogenesis after the knockdown of PTSMAD4, we analyzed the KEGG results and discovered multiple signaling pathways involved in spermatogenesis. Studies have shown that in the Wnt signaling pathway, β-catenin enters the nucleus under the regulation of Wnt signaling molecules to activate the expression of related genes, promoting the formation of mature sperm and participating in the spermatogenesis process of the Chinese mitten crab [53]. The regulated Wnt/β-catenin can maintain the spermatogenesis process in mice [8]. The phosphatidylinositol 3-kinase-protein kinase pathway is one of the most important regulatory mechanisms in mammalian cells, mainly due to it regulating the proliferation, survival, and apoptosis of cell lines to directly or indirectly maintain spermatogenesis [7]. In the GnRH signaling pathway, the injection of GnRH homologs can shorten the ovarian maturation period of the whiteleg shrimp [54], and exogenous GnRH subtypes can increase the sperm production of the swimming crab P. trituberculatus [55]. This study explored the function of PTSMAD4 during the spermatogenesis in P. trituberculatus. Based on transcriptome data and existing studies on other species, we speculated that PTSMAD4 regulated gene expression during spermatogenesis and may be cross-talked with other signal pathways—for example, the Wnt signaling pathway, the PI3K-Akt signaling pathway. This study further improved the knowledge of the mechanism of spermatogenesis in crustaceans and laid a theoretical foundation for better species selection.

## 4. Materials and Methods

### 4.1. Preparation of Animals and Samples

The swimming crabs *P. trituberculatus* (n = 30) used in this experiment were purchased from Qixin Aquaculture Farm (Ningbo, China) and were acclimated in the laboratory for one week before sampling. The crabs were anesthetized on ice, and tissues, including the testes, vas deferens, heart, hepatopancreas, gills, and muscle, were dissected out. These tissues were snap-frozen in liquid nitrogen and stored at −80 °C. Additionally, a portion of the testis tissue was fixed in 4% paraformaldehyde for 2 h, rinsed twice with PBS, and dehydrated overnight in 1 mL of 0.5 M sucrose solution. The tissue was then embedded in an OCT compound (SAKURA, Torrance, CA, USA), frozen at −20 °C, and sectioned using an HM525NX cryostat (Thermo Fisher Scientific, Shanghai, China). The frozen sections were stored at −80 °C. The New Zealand rabbits used in this laboratory were purchased from the Experimental Animal Center of Ningbo University and acclimated for one week before the immunization injections began.

### 4.2. Extraction of Total RNA and Reverse Transcription to cDNA

Tissue samples (0.3 mg) were placed in 1 mL of RNA-Solv Reagent (Omega, Guangzhou, China) and homogenized on ice using a T10 basic S25 homogenizer (IKA, Hessen, Germany). The samples were then vortexed for 15 s and left to stand on ice for 5 min. Next, 200 μL of precooled chloroform (Guoyao, Beijing, China) was added, and the mixture was vigorously vortexed for 30 s and then left to stand on ice for 3 min. The samples were centrifuged at 4 °C at 12,000× *g* for 15 min. The supernatant was collected, and an equal volume of isopropanol (Guoyao, Beijing, China) was added. After vigorous vortexing for 10 s and standing for 2 min, the samples were left at −20 °C for 30 min before being centrifuged at 4 °C at 12,000× *g* for 10 min to obtain the RNA pellet. The pellet was washed with 75% ethanol (Guoyao, Beijing, China) and then dissolved in 20 μL of DEPC water. The RNA quality was assessed by gel electrophoresis, and the RNA concentration was measured using a NanoDrop 2000 (Thermo Fisher Scientific, Shanghai, China). The cDNA was synthesized from RNA using the HiFiScript cDNA Synthesis Kit (Kangwei Century, Hangzhou, China) and stored at −80 °C.

### 4.3. Cloning of Full-Length cDNA Sequences

The PTSMAD4 gene fragment was obtained through database alignment at the National Center for Biotechnology Information (www.ncbi.nlm.nih.gov, accessed on 9 January 2023). The primers were designed using Primer Premier 5 (see Table 1). The PCR program was as follows: 94 °C for 5 min, 8 cycles of 94 °C for 30 s, 59 °C with a decrease of 0.5 °C per cycle for 30 s, and 72 °C for 1 min, followed by 27 cycles of 94 °C for 30 s, 55 °C for 30 s, and 72 °C for 1 min, with a final extension at 72 °C for 10 min. After gel electrophoresis, the PCR products were excised and purified using an agarose gel DNA recovery kit (Bioteke, Wuxi, China). The DNA fragments were ligated into the pMD19-T Vector (Takara, Beijing, China) using a PCR program of 16 °C for 8 h. The ligation products were transformed into DH5α competent cells (ice bath for 30 min, heat shock at 42 °C for 90 s, and ice bath for 3 min). After adding 800 μL of antibiotic-free LB medium, the cells were incubated at 37 °C for 2 h. Then, 50 μL of the bacterial culture was spread on AMP+ antibiotic plates and incubated overnight at 37 °C in an HPX-9082MBE incubator (Shanghai Boshen Medical Biological Instrument Co., Ltd., Shanghai, China). Positive monoclonal colonies were picked, and after bacterial identification, the samples were sent to Youkang Biotech Co., Ltd. (Hangzhou, China) for sequencing. All primers used in this study are listed in Table 1.

### 4.4. Semi-Quantitative PCR

Semi-quantitative primers were designed using Primer Premier 5 (see Table 1). The cDNA from various tissues of the swimming crab (testes, vas deferens, hepatopancreas, gills, muscle, and heart) was used as a template, with β-actin as the internal control. Three crabs were used per sample, with three replicates per crab, for semi-quantitative PCR. The PCR program was as follows: 94 °C for 30 s, followed by 32 cycles of 94 °C for 30 s, 60 °C for 30 s, and 72 °C for 30 s, with a final extension at 72 °C for 10 min. PCR products were subjected to gel electrophoresis, and the gray values were extracted using Image J V1.8.0.112. Column charts were drawn using Primer 8.0 software.

### 4.5. Production of Specific Antibodies

#### 4.5.1. Prokaryotic Expression

Prokaryotic expression primers were designed using Primer Premier 5 (see Table 1), with restriction sites and protective bases added to the 3′ ends of the forward and reverse primers. The restriction sites were BamH I and Xho I (Takara, Beijing, China). The PCR program was as follows: 94 °C for 5 min; 35 cycles of 94 °C for 30 s, 60 °C for 30 s, and 72 °C for 1 min, with a final extension at 72 °C for 10 min. The PTSMAD4 gene prokaryotic expression fragment was amplified and the DNA fragment was recovered (as described in Section 4.3). Subsequently, the DNA fragment and pET-28a(+) plasmid were double-digested (30 °C for 6–8 h; 37 °C for 2 h). Gel electrophoresis was used to check the sizes of the DNA and pET-28a(+) plasmid fragments. The digested DNA fragment and pET-28a(+) plasmid were ligated using T4 DNA Ligase (Takara, Beijing, China) at 16 °C for 8 h. The ligation products were transformed into DH5α competent cells (as described in Section 4.3). After adding 800 μL of antibiotic-free LB medium and incubating it at 37 °C for 2 h, 50 μL of the bacterial culture was spread onto Kana+ antibiotic plates and incubated overnight at 37 °C. Positive monoclonal colonies were picked for bacterial identification and sent to Youkang Biotech Company for sequencing. The sequenced plasmids were then transformed into Transetta (DE3) expression bacteria (as described in Section 4.3). After adding 800 μL of antibiotic-free LB medium and incubating at 37 °C for 2 h, 50 μL of the bacterial culture was spread on Kana+ antibiotic plates and incubated overnight at 37 °C. Positive monoclonal colonies were picked for bacterial identification and sent to Youkang Biotech Company for sequencing. A small-scale induction was then performed. When the bacterial culture OD value reached 0.4–0.8, 1 μL of IPTG (SoluLab, Beijing, China) was added to induce expression, and another 1 mL of bacterial culture served as a control. After incubation at 37 °C for 6 h, the cells were centrifuged at 5000× *g* for 1 min, and the supernatant was discarded. The pellet was washed twice with 50 μL of 1× PBS. The bacterial cells were resuspended in 50 μL of 1× PBS, and 12.5 μL of 5× SDS loading buffer (Beyotime, Shanghai, China) was added. After vortex mixing, the mixture was denatured at 100 °C for 10 min. The denatured proteins were subjected to SDS-PAGE electrophoresis (160 V for 45 min), followed by staining with Coomassie Brilliant Blue (Beyotime, Shanghai, China) for 20 min and incubation at 100 °C for 10 min. The proteins were then induced on a large scale. Inclusion body proteins were purified according to the instructions of a His-tag protein purification kit (Kangwei Century, Hangzhou, China). The concentration of the recombinant protein was measured using a Bradford assay kit (Beyotime, Shanghai, China).

#### 4.5.2. Antigen Immunization

The purified recombinant protein was used for periodic antigen injections in New Zealand rabbits over a period of 5 weeks. The first subcutaneous injection was 600 μg of recombinant protein, which was mixed with an equal volume of Freund’s complete adjuvant and emulsified thoroughly. Subsequent injections (2–5) were 300 μg of recombinant protein mixed with an equal volume of Freund’s incomplete adjuvant and emulsified thoroughly. One week after the fifth injection, the rabbits were anesthetized, and blood was collected and immediately processed. The blood was left at 4 °C overnight, followed by centrifugation at 4 °C at 12,000× *g* for 30 min. The supernatant was collected to obtain the desired antibodies, which were then stored at −80 °C.

#### 4.5.3. Extraction of Total Protein

We extracted the total protein from the testis of three crabs (n = 3). We thoroughly mixed 1 mL of RIPA cracking buffer (Beyotime, Shanghai, China) with 10 μL PMSF (SoluLab, Beijing, China). Tissue samples (0.3 g) from the swimming crab testes were added and homogenized using a T10 basic S25 homogenizer for 2 min (15 s on, 10 s off), and then left to stand on ice for 30 min. The samples were centrifuged at 4 °C at 12,000× *g* for 15 min, and the supernatant was collected as the required protein, which was stored at −80 °C. For protein denaturation, 100 μL of the supernatant was mixed with 25 μL of 5× SDS loading buffer, vortexed, and denatured at 100 °C for 10 min. After cooling, the mixture was stored at −20 °C.

#### 4.5.4. Western Blot (WB) Validation of Antibodies

Future PAGE precast gels (ACE, Changzhou, China) were used to separate proteins of different molecular weights. We extracted the total protein of the testis tissue of the swimming crab (n = 3). Each well was loaded with 20 ng of the total protein and run at a constant voltage of 160 V for 45 min. Six sheets of filter paper of the same size as the protein gel and one 0.45 μm PVDF membrane (SoluLab, Beijing, China) (0.5 cm larger than the filter paper) were cut. The filter paper was soaked in rapid transfer buffer (Shanghai Qi Chun, Beijing, China), and the PVDF membrane was activated in methanol for 30 s, shaken in pure water for 3 min, and then placed in the rapid transfer buffer. After 30 min of transfer at 400 mA, the PVDF membrane was blocked in 5% skim milk for 2 h. The membrane was washed with 0.1% TBST for 5 min and then incubated with the homemade anti-PTSMAD4 rabbit antibody (1:500 dilution) and anti-β-actin rabbit antibody (Beyotime, Shanghai, China) (1:1500 dilution) at 4 °C overnight. The membrane was washed with 0.1% TBST four times, each for 10 min. HRP-conjugated goat anti-rabbit antibody (Beyotime, Shanghai, China) (1:1500 dilution) was used for a 2 h incubation at 37 °C, followed by four washes with 0.1% TBST for 10 min each. WesternBright ECL reagent (Advansta, San Jose, CA, USA) was added, and the membrane was developed and photographed using a Tanon 5200 automatic chemiluminescence imaging analysis system (Tanon, Shanghai, China).

### 4.6. Immunofluorescence

Frozen sections stored at −80 °C were taken out and left to dry at room temperature for 10–15 min. The sections were permeabilized with 0.3% PBST for 20 min at room temperature. The tissue was blocked with 5% BSA-PBS for 2 h at room temperature, washed with 0.1% PBST for 5 min, and then incubated with the antibody dilution solution (homemade anti-PTSMAD4 rabbit antibody; 1:50 dilution). After overnight incubation at 4 °C, the sections were washed three times with 0.1% PBST for 10 min each. The fluorescent secondary antibody (Alexa Fluor 488 conjugated goat anti-rabbit IgG (H + L) (Beyotime, Shanghai, China), 1:500 dilution) was applied and incubated at room temperature for 2 h. After six washes with 0.1% PBST for 10 min each, DAPI (Beyotime, Shanghai, China) was applied for 5 min, followed by the addition of an anti-fluorescence quenching agent (Beyotime, Shanghai, China). A cover slip was placed, and the slide was sealed with nail polish. After air-drying at room temperature, the slides were stored at 4 °C in the dark and observed and photographed using an LSM880 confocal laser scanning microscope (Carl Zeiss, Oberkochen, Germany).

### 4.7. Preparation of dsRNA and In Vivo Interference in Swimming Crabs

#### 4.7.1. Preparation of dsRNA

To further investigate the function of PTSMAD4 in spermatogenesis, primers were designed based on the full-length cDNA sequence of PTSMAD4 using Primer Premier 5 (see Table 1). The dsRNA was synthesized according to the instructions of the TranscriptAid T7 High Yield Transcription Kit. Gel electrophoresis was used to check the size of the synthesized fragment (462 bp), and the concentration of dsRNA was measured using a NanoDrop 2000. The dsRNA was diluted to 2 μg/μL with 1× PBS and stored at −80 °C.

#### 4.7.2. In Vivo Interference in Swimming Crabs

Healthy male swimming crabs were selected, and nine crabs were placed in each group, with two groups in total: the PTSMAD4 interference group (n = 9) and control group (n = 9). The injection cycle was repeated five times at 48 h intervals. Twenty-four hours after the fifth injection, the crabs were dissected on ice, and tissues, including the testes, vas deferens, heart, gills, muscle, and hepatopancreas, were collected and stored at −80 °C.

### 4.8. qPCR

Primer Premier 5 was used to design specific primers for PTSMAD4 (see Table 1), with β-actin as the reference gene. The diluted cDNA (30 times) was used as a template, and qPCR reactions were performed using the 2× RealStar Green Fast Mixture kit. The qPCR program was as follows: 95 °C for 2 min, followed by 40 cycles of 95 °C for 15 s, 60 °C for 30 s, and 72 °C for 30 s. The PBS group served as the control group. The interference efficiency of *PTSMAD4* mRNA and the expression of related genes were analyzed using the ∆∆Ct method.

### 4.9. Library Construction and Sequencing Dynabeads

Oligo magnetic beads were used to enrich mRNA with a polyA structure from the total RNA. Divalent cations were used to fragment the mRNA into short pieces, which were reverse transcribed into the first cDNA strand using SuperScript™ II Reverse Transcriptase (Takara, Beijing, China). The second cDNA strand was synthesized using the first cDNA strand as a template. A-tails were added to the cDNA to prepare for the ligation of sequencing adapters. PCR amplification was performed to enrich the cDNA library. After constructing the cDNA library, all samples were sequenced using the Illumina NovaseqTM 6000 sequencing platform (LC-Bio Technologies, Hangzhou, China).

The raw data were filtered using Cutadapt to remove inaccurate sequences, and clean data were obtained. Hisat 2 was used to align the Clean Data with the reference genome, and the alignment results were used by StringTie to reconstruct transcripts and calculate the gene expression levels in the samples. Differentially expressed genes were selected with a threshold of FC ≥ 2 or FC ≤ 0.5 and a *q*-value < 0.05, and GO, KEGG, and other enrichment analyses were performed on the differential genes. The significant enrichment analysis of GO classified the significantly expressed differential genes into various terms. The raw data were uploaded to NCBI. The BioProject ID is PRJNA1155623.

## 5. Conclusions

In this study, we first cloned and obtained the full-length cDNA sequence of PTSMAD4 and conducted bioinformatics analysis on it. The analysis results indicate that the structural domains of the PTSMAD4 protein are highly conserved. *PTSMAD4* mRNA is widely expressed in various tissues, with particularly high expression levels in the testes. During spermatogenesis, the PTSMAD4 protein is expressed in the cytoplasm at different developmental stages, suggesting that PTSMAD4 may be involved in the process of meiosis. We also observed co-localization of PTSMAD4 with PTSMAD2 proteins in spermatocytes and early spermatids, whereas no co-localization was found in mature sperm. After knocking down PTSMAD4 using RNA interference technology, we found that the signals of PTSMAD4 and PTSMAD2 during all stages of spermatogenesis were weakened, and co-localization was also reduced. We measured the protein abundance of PTSMAD2 after knockdown PTSMAD4, and the results showed that the protein abundance of PTSMAD2 decreased significantly. These results suggest that PTSMAD4 may cooperate with PTSMAD2 to regulate gene expression during the meiotic phase and the early stages of sperm formation, thereby participating in the spermatogenesis process. Furthermore, by comparing the transcriptome data before and after interference, we found that genes and signaling pathways related to spermatogenesis were enriched after PTSMAD4 interference. Based on the above results, we speculate that PTSMAD4 may be involved in the process of spermatogenesis in swimming crabs (*P. trituberculatus*) through signal transduction.

## Figures and Tables

**Table 1:** Primer sequence used in this experiment.

Primer	Sequence (5′-3′)	Purpose
PTSMAD4-F	CAAGTGTGACTCGGTGTGC	PCR-cloning
PTSMAD4-R	GCGGGATGGTGTGTAGAA	PCR-cloning
PTSMAD4-3′F1	CCCTCCGTCACTGTGGATGGCTAT	3′RACE
PTSMAD4-3′F2	CTGAGCAGAGTGAGAGGGCAAGGC	3′RACE
PTSMAD4-3′F3	AAGGAAACCCCCTGCTGGATTGAG	3′RACE
PTSMAD4-5′R1	GACGGCACGGAGTCGGTGAAGAAT	5′RACE
PTSMAD4-5′R2	CGCTGAGGCGGAAACTGTCTGG	5′RACE
PTSMAD4-5′R3	GGGACACGACACGCTCATAGTGGTAG	5′RACE
PTSMAD4-BF	CGTGCCATTGAAAGTCTCGTC	qPCR
PTSMAD4-BR	CCCACTCACCTCACACTCCATA	qPCR
β-actin-F	CATCAAGGAAAAGTTGTGCTACG	qPCR
β-actin-R	CCATACCCAGGAAGGAAGGC	qPCR
PTSMAD4-YF	CGCGGATCCCAGGTAGGGGAGACATTCAAGGT	Antibody
PTSMAD4-YR	CCGCTCGAGCTCAATCCAGCAGGGGGTT	Antibody
PTSMAD4-WF	GGAGTTCTGGTGTTCTATCGCA	RNAi
PTSMAD4-WR	GAGCGGGATGGTGTGTAAAAC	RNAi
PTSMAD4-dsT7F	TAATACGACTCACTATAGGGCTGTGGATGGCTATGTGGACCC	RNAi
PTSMAD4-dsR	TCAGGCGTAGGATACAGAGGCG	RNAi
PTSMAD4-dsF	CTGTGGATGGCTATGTGGACCC	RNAi
PTSMAD4-dsT7R	TAATACGACTCACTATAGGGTCAGGCGTAGGATACAGAGGCG	RNAi
PTSMAD4-qF	CTGAGCAGAGTGAGAGGGCA	qPCR
PTSMAD4-qR	AATACAGGCACACGGGTAAATC	qPCR
bdh-F	AACTTCAATGCTGCGACCCA	qPCR
bdh-R	GCTTGCCGTAATCAGACCTCAC	qPCR
Sod1-F	CCCTCCGTCACTGTGGATGGCTAT	qPCR
Sod1-R	CTGAGCAGAGTGAGAGGGCAAGGC	qPCR
PLD beta1-F	GACCCAGCACCCAAGCAT	qPCR
PLD beta1-R	CAAAGCCGCACTCACCCT	qPCR
ste20-F	CATCCTTACCGCTCGCTGG	qPCR
ste20-R	GAAAGTGTCGTCCCCGCA	qPCR
β-actin-F	CATCAAGGAAAAGTTGTGCTACG	qPCR
β-actin-R	CCATACCCAGGAAGGAAGGC	qPCR
mys-F	AGACCTCCTCTTTTGGCTTGC	qPCR
mys-R	GTGATGGCGTTGGTGCTGT	qPCR
CenG1A-F	TCTGGTCCAATGCCAATGTGA	qPCR
CenG1A-R	GGCTAAGAGCAGGGGTGTGAG	qPCR
Bbox1-F	CGTCCTAACCGATGCTCCC	qPCR
Bbox1-R	ACCCTTGCCCTCAAACTGTG	qPCR
Rab-35-F	AACGCTTCCGAACTATCACATC	qPCR
Rab-35-R	ACAGTCATTCTTATTCCCCACG	qPCR
Ago4-F	CCATCCACCAGGGGCTTCT	qPCR
Ago4-R	CTGCCGTGACGCAATCTCC	qPCR
mov10b.1-F	GGACCAGATGCTGATTCGCT	qPCR
mov10b.1-R	TTCTTCACCCACGCCACC	qPCR

## Data Availability

Data are contained within the article and Supplementary Materials.

## Supplementary Materials

The following supporting information can be downloaded at https://www.mdpi.com/article/10.3390/ijms252313126/s1.
