# Supplementary material for: Functional Study of PTSMAD4 in the Spermatogenesis of the Swimming Crab Portunus trituberculatus
Source: Int J Mol Sci. 2024 Dec 6;25(23):13126. doi: 10.3390/ijms252313126 (PMC11642582; doi:10.3390/ijms252313126)
Supplement: Supplementary file 1 [file ijms-25-13126-s001.zip › ijms-3216988-supplementary.pdf]

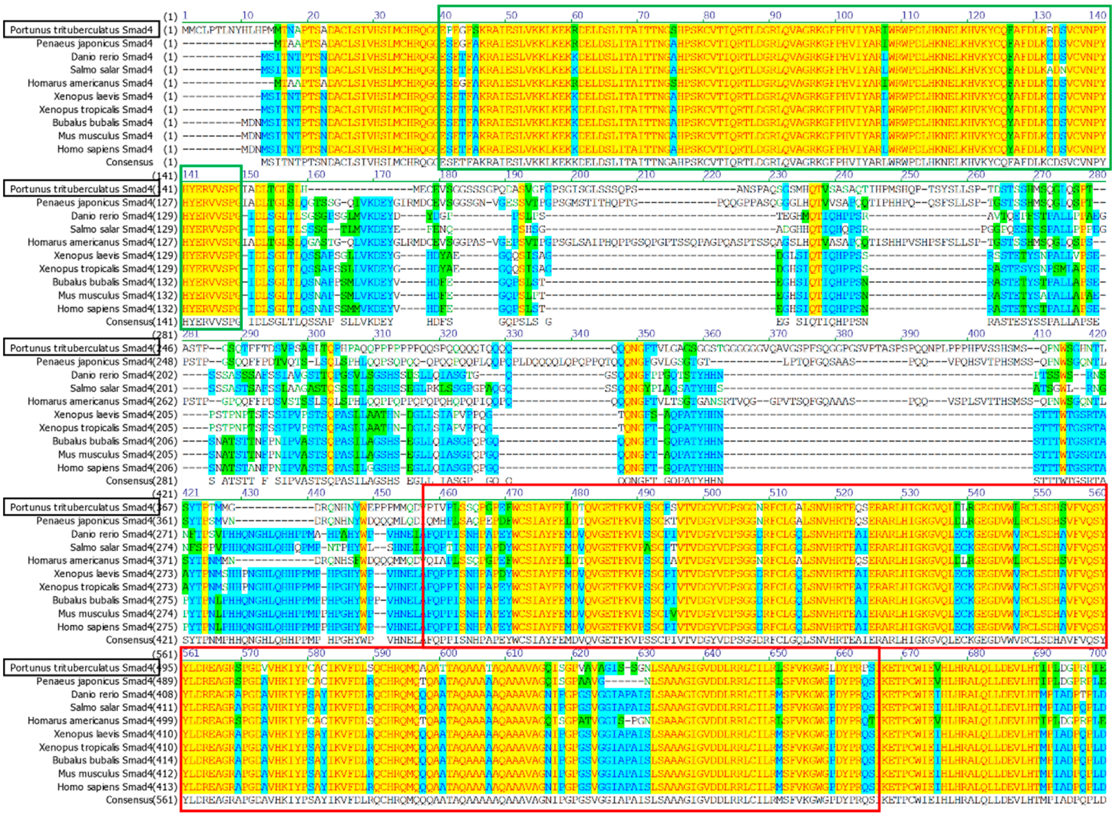

Supplementary Figure S1. Amino acid multiple sequence alignment of PTSMAD4. The black frame represents the swimming crab P. trituberculatus, The green boxes are MH1 domains, The red box is the MH2 do-main.

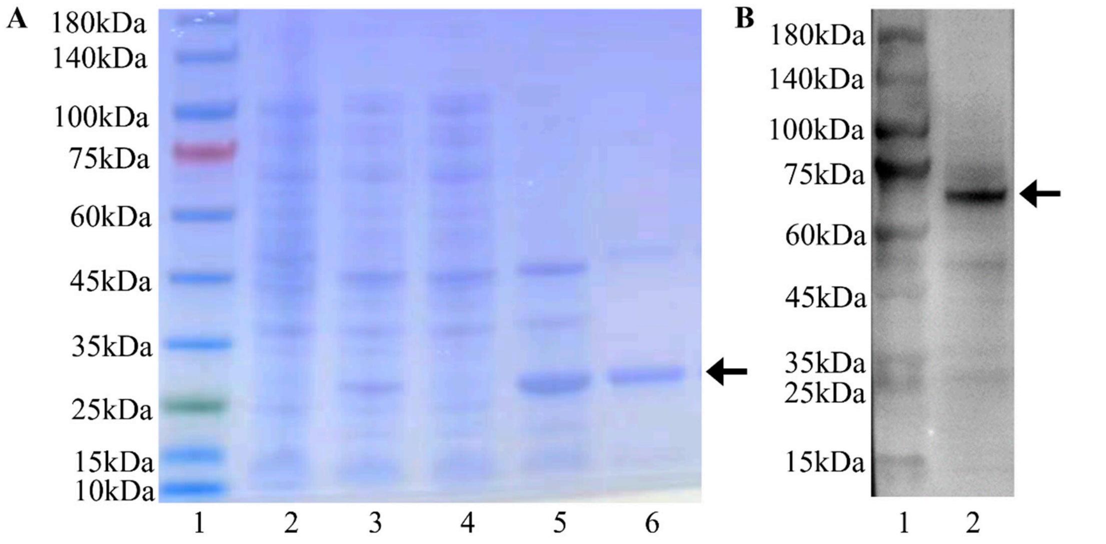

Supplementary Figure S2. (A) Coomassie Brilliant Blue staining to examine the prokaryotically expressed recombi-nant PTSMAD4 protein. Lane 1: Protein marker. Lane 2: Uninduced expressed protein; Lane 3: Induced expressed protein; Lane 4: Supernatant of induced expressed protein; Lane 5: Precipitate of induced expressed protein; Lane 6: Purified recombinant PTSMAD4 protein; (B) Validation of the polyclonal rabbit antibody against PTSMAD4.
